# Oxidative stress and the altered reaction to it in Fabry disease: A possible target for cardiovascular-renal remodeling?

**DOI:** 10.1371/journal.pone.0204618

**Published:** 2018-09-27

**Authors:** Verdiana Ravarotto, Gianni Carraro, Elisa Pagnin, Giovanni Bertoldi, Francesca Simioni, Giuseppe Maiolino, Matteo Martinato, Linda Landini, Paul A. Davis, Lorenzo A. Calò

**Affiliations:** 1 Department of Medicine, Nephrology, University of Padua, Padua Italy; 2 Department of Medicine, Hypertension, University of Padua, Padua Italy; 3 Clinical Research Unit, Padua University Hospital, Padua, Italy; 4 Medical Affairs Sanofi-Genzyme, Milan, Italy; 5 Department of Nutrition, University of California, Davis, United States of America; International University of Health and Welfare, School of Medicine, JAPAN

## Abstract

**Background:**

Fabry disease is characterized by deficient expression/activity of α-GalA with consequent lysosomal accumulation in various organs of its substrate Gb3. Despite enzyme replacement therapy, Fabry disease progresses with serious myocardial, cerebral and renal manifestations. Gb3 accumulation may induce oxidative stress (OxSt), production of inflammatory cytokines and reduction of nitric oxide, which may impact on Fabry disease’s clinical manifestations.

**Methods:**

OxSt status was characterized in 10 patients compared with 10 healthy subjects via protein expression of p22^phox^, subunit of NADH/NADPH oxidase, (Western blot), Heme oxygenase (HO)-1 levels (ELISA), antioxidant/anti-inflammatory, lipid peroxidation as malondialdehyde (MDA) production (colorimetric assay), phosphorylation state of Extracellular Signal Regulated Kinase (ERK)1/2 and Myosin Phosphatase Target Protein (MYPT)-1 (Western blot), marker of Rho kinase activation, both involved in OxSt signaling. Cardiac left ventricular (LV) mass was also evaluated (M-mode echocardiography).

**Results:**

LV mass was higher in Fabry’s males (123.72±2.03SEM g/m^2^) and females (132.09±6.72g/m^2^). p22^phox^ expression was also higher in patients (1.04±0.09 d.u. vs 0.54±0.05 d.u. p<0.01) as well as MDA levels (54.51±3.97 vs 30.05±7.11 nmol/mL p = 0.01) while HO-1 was reduced (8.84±0.79 vs 14.03±1.23 ng/mL, p<0.02). MYPT-1’s phosphorylation was increased in patients (0.52±0.11 d.u. vs 0.03±0.08 d.u., p<0.01) while phosphorylation of ERK1/2 was reduced (0.91±0.08 d.u. vs 1.53±0.17 d.u., p = 0.004).

**Conclusions:**

This study documents OxSt activation and the altered reaction to it in Fabry patients. Cardiac remodeling, Rho kinase signaling activation and reduction of protective HO-1 might suggest that, in addition to enzyme replacement therapy, OxSt inhibition by either pharmacological or nutritional measures, is likely to prove useful for the prevention/treatment of Fabry patients’ cardiovascular-renal remodeling.

## Introduction

Fabry disease (OMIM 301500) is an X-linked storage disorder with mutations affecting the α-galactosidase A gene (GLA, α-GalA, E.C. 3.2.1.22) which encodes for α-GalA. This disease results from the progressive accumulation of globotriaosylceramide (Gb3), its deacetylated form (Lyso-Gb3) and other glycosphingolipids in lysosomes due to insufficient activity of the lysosomal enzyme α-galactosidase A. Fabry disease presents in the heterozygous form in women and hemizygous in men [1]. However due to a wide range of symptoms (e.g. rheumatic fever, abdominal pain, angiokeratoma, hearing loss, acroparesthesias, corneal opacity, etc.), early stage Fabry disease is difficult to recognize [2]. Fabry disease’s most severe manifestations are mainly vascular, leading to cerebrovascular disease, cardiac disease, and or progressive renal failure. The current standard treatment for Fabry disease is enzyme replacement therapy (ERT) by intravenous administration of an enzyme replacement with either of two enzymes, agalsidase-alfa (Shire HGT) or agalsidase-beta (Genzyme Corp). Oral treatments using molecules targeted to stabilize the conformation of the enzyme are undergoing clinical trials, although they are effective for only few GLA mutations [3]. ERT results in the clearance of Gb3 inclusions from the capillary endothelium of the kidneys as well as the progression of lysosomal storage in other cell types such as smooth muscle and myocardium cells. Unfortunately, since the trials on agalsidase-alfa and agalsidase-beta have been authorized by the European Medicines Agency (EMA), no clear evidence has emerged that ERT alters the natural course of Fabry disease-associated cardiac or cerebrovascular disease or nephropathy, in particular when late diagnosis is the case. This suggests that these adverse outcomes are not entirely dependent on the simple accumulation of glycosphingolipids [4].

Oxidative stress (OxSt) is central in cardiovascular-renal remodeling that represents the most common cause for excess morbidity and mortality in high cardiovascular risk patients [5,6]. OxSt in Fabry disease has been assessed only in terms of elevated plasma levels of proinflammatory cytokines and the molecular mechanisms causing the severe phenotype in Fabry disease remain unclear [7]. Given the elevated proinflammatory cytokines, OxSt related proteins and OxSt signaling appear likely to play an important role in the processes involved in the pathophysiology of Fabry disease [8–10] and a better understanding of the biological processes that lead to endothelial dysfunction and disease progression in Fabry disease may suggest new treatment strategies in order to lessen the high morbidity found in Fabry disease patients.

We aimed to investigate the OxSt state in Fabry disease patients currently undergoing ERT using a molecular biology approach. In the current study, we evaluated the cardiac remodeling in Fabry disease through the assessment of left ventricular hypertrophy (LVH) via echocardiographic determination of LV mass. In concert with that, OxSt signaling was evaluated through assessing several known markers of OxSt. In particular we assessed the protein levels of p22^phox^, a subunit of NADPH oxidase essential for the production of superoxide anions alongside the oxidative degradation of lipids by measuring the production of reactive aldehydes as malondialdehyde (MDA) [11]. Moreover, we evaluated the expression of heme-oxygenase inducible isoform 1 (HO)-1, antioxidant, antiinflammatory and atiapoptotic protein [12]. In addition, the phosphorylation state of both the myosin phosphatase target protein (MYPT)-1 and extracellular signal-regulated kinase 1/2 (ERK 1/2) were also determined. MYPT-1 phosphorylation is a marker of Rho kinase activation that modulates the phosphorylation state of the regulatory chain of myosin II leading to both vasoconstriction and cardiovascular-renal remodeling and the phosphorylation state of the extracellular signal-regulated kinase (ERK)1/2, a member of the MAPK family, which is an effector protein of OxSt for cardiovascular-renal remodeling [8,13,14]. In addition, as high LysoGb3 has been recently shown in an in vitro model to be associated with increased intracellular Adenosine-3’,5’-cyclic monophosphate (cAMP) and elevated cAMP may induce a reduction in the phosphorylation of ERK1/2 via protein kinase A (PKA) activity [15,16], we have measured the plasma level of cAMP in Fabry patients and compared them to controls.

## Materials and methods

### Patients

All procedures in this study were performed studies with the approval of the Ethical Committee University Hospital of Padua (CESC) with protocol number 3748, 25/2/2016. Subjects have given their informed written consent.

We studied a cohort of 10 Fabry disease patients (five males and five females, 28–70 years old) followed at our Nephrology Unit. Lyso Gb3 levels have been evaluated by Centogene-The Rare Disease Company (Rostock, Germany) on a dried blood spot by high performance liquid chromatography and tandem mass spectrometry alongside the assessment of GLA gene mutations (reference accession number NM_000169.2) by NGS Illumina sequencing. Lyso Gb3 levels and the genetic characterization of Fabry disease patients of the present study are shown in Table 1.

**Table 1:** Genetic characterization and plasma levels of Lyso Gb3 assessed in Fabry disease patients.

Patient nr.	Gender	GLA Mutation	Lyso Gb3 ng/mL
#1	male	c.103G>C	28.6
#2	female	c.835C>A	9
#3	female	c.835C>A	7.8
#4	female	c.835C>A	6.9
#5	female	c.779G>A	9.8
#6	male	c.779G>A	7.9
#7	mele	c.811G>A	26
#8	male	c.773G>T	12
#9	female	c.723dupT	18
#10	male	c.641C>T	9

Plasma Lyso Gb3 normal levels are: ≤1.8 ng/mL

Among the patients, one was under chronic dialysis and one underwent renal transplantation. Dialysis patient was under chronic dialysis treatment with 210–240 min, three times a week with low-flux bicarbonate-dialysis with ultrapure dialysate, using a polysulphone dialyser 1.8 m^2^ bicarbonate-dialysis. The dialysis patient was treated with epoetin (EPO, 10,000 UI/week) along with vitamin D (1.25 dihydroxyvitamin D3, 25 mg every 2 days) and calcium supplements. Fabry disease patients were treated with either agalsidase-beta 1 mg/kg every two weeks (N = 5) or agalsidase-alpha 0.2 mg/kg every two weeks (N = 5). Patients' blood pressure ranged from 134/84 to 154/92 mmHg. The three hypertensive patients were under antihypertensive treatment (see Table 2). None of the patients was a smoker and under lipid lowering treatment. Patients characteristics are shown in Table 2.

**Table 2:** Patients characteristics.

Patient nr.	#1	#2	#3	#4	#5	#6	#7	#8	#9	#10
**Age**	51	43	70	68	65	43	43	28	51	42
**SBP/DBP mmHg**	134/84	140/90	138/88	135/85	145/92	130/85	140/89	150/89	130/90	154/92
**Heart Rate bpm**	80	83	78	75	88	79	81	92	85	94
**BMI**	24.9	21.4	21.7	21.3	21.5	24.3	24.5	23.7	24.8	23.5
**Glycemia mmol/L**	5.22	4.99	3.99	4.20	3.98	5.35	5.12	4.89	5.55	4.77
**HbA1C mmol/L**	40.1	35.7	32.5	37.6	27.9	39.2	41.5	37.2	41.3	35.1
**Total Cholesterol mmol/L**	4.75	4.51	5.06	4.99	4.69	4.89	5.12	4.56	5.18	5.15
**HDL mmol/L**	1.22	1.62	1.35	1.10	1.25	1.13	1.04	1.11	1.02	1.03
**LDL mmol/L**	2.88	2.79	3.34	2.37	2.56	2.98	3.20	3.09	3.35	3.29
**Triglycerides mmol/L**	1.59	1.23	1.42	1.35	1.28	1.32	1.55	1.48	1.69	1.58
**GFR mL/min/1.73m**^**2**^	87	87	85	90	83	89	haemodialysis	86	72	85
**ERT therapy** **Algasidase-**	-alpha 0.2 mg/Kg	- alpha 0.2 mg/Kg	-beta 1 mg/Kg	-beta 1 mg/Kg	- alpha 0.2 mg/Kg	-beta 1 mg/Kg	-beta 1 mg/Kg	- alpha 0.2 mg/Kg	- alpha 0.2 mg/Kg	-beta 1 mg/Kg
**Additional Treatment**							EPO 10,000UI/week; Vitamin D; calcium; amlodipine 10mg doxazosin 2mg		renal transplanted; amlodipine 10mg; doxazosin 2mg	amlodipine 10mg; ramipril 5mg

Ten healthy subjects (four males and six females, 29–50 years old) were recruited from the staff of the Department of Medicine-DIMED, at the University of Padova and used as control.

All the subjects abstained from food, alcohol and caffeine-containing drinks for at least 12 hours before blood withdrawal. Blood was collected for each subject early in the morning and for Fabry patients blood sampling was done always before the enzyme infusion, after two weeks from the previous ERT infusion. Informed consent was obtained from all the study participants and the study protocol was approved by institutional authorities.

### Preparation of mononuclear cells

The blood samples were processed the same day immediately after the collection. Plasma fractions were removed by centrifugation from 35 ml of EDTA anticoagulated blood at room temperature and immediately stored at -80°C for the following procedures. Peripheral blood mononuclear cells (PBMCs) were isolated at room temperature by Lympholyte-H gradient (Cedarlane, Hornby, Ontario, CA). Total protein extracts were obtained by cell lysis using an ice-cold buffer (Tris-HCL 20mmol/l, NaCl 150mmol/l, EDTA 5.0mmol/l, Niaproof 1.5%, Na3VO4 1.0mmol/l, SDS 0.1%) added with protease inhibitors (Roche Diagnostics, Mannheim, DE) and phosphatase inhibitors (Phosphatase Inhibitor Cocktail 1, Sigma-Aldrich, Saint Louis, MI, USA). The protein solution was immediately stored at -80°C for the following procedures. Once collected, all the samples were placed on ice and underwent a sonication cycle. Samples were centrifuged and the supernatant solution containing proteins extracts were collected. Finally, the protein concentration was evaluated by BCA protein assay (Pierce, Rockford, Illinois, USA) at room temperature. The samples were stored at -80°C until the experiment procedures were performed.

### p22^phox^, ERK1/2 and MYPT-1 Western blot analysis

Protein expression of p22^phox^ and phosphorylation state of MYPT-1 and ERK1/2 were assessed by Western blot analysis as previously reported [14]. Briefly, protein extracts of samples from patients and controls were separated by SDS-PAGE in Tris pH 8.3 as 10 samples per gel with a molecular weight marker (Full-Range Rainbow Molecular Weight Marker, GE Healthcare, Amersham Pl, UK). In each gel there was an equal amount of samples from patients and controls (5 patients and 5controls). Subsequently, proteins were transferred on nitrocellulose membranes using a Hoefer TE 22 Mini Tank Transphor Unit (Amersham Pharmacia Biotech, Uppsala, SE) in a buffer composed by: 39mmol/l glycine, 48mmol/l Tris base, 0.037% SDS (electrophoresis grade), and 20% methanol. The number of gel prepared was equal to the number of the targeted proteins. After the blocking with no-fat milk solution (5% in Tween-PBS), each membrane was incubated overnight with a primary polyclonal antibody for the detection of a specific protein: anti-p22^phox^ (Santa Cruz Biotechnologies, Santa Cruz, CA, USA); anti-ERK1/2 (Cell Signaling technology, Danvers, MA, USA); anti-phospho ERK1/2 (Cell Signaling technology, Danvers, MA, USA); anti-MYPT-1 (Cell Signaling technology, Danvers, MA, USA); anti-phospho MYPT-1 (Cell Signaling technology, Danvers, MA, USA). To normalize the result, antimonoclonal antibodies were used to detect α-tubulin (anti-α-tubulin, Santa Cruz Biotechnologies, Santa Cruz, CA, USA), GAPDH (anti-GAPDH, Merk Millipore, Darmstadt, DE) and β-actin (anti-β-actin, Santa Cruz Biotechnologies, Santa Cruz, CA, USA) as houkeeping in each membrane. Specific secondary antibodies were HRP-conjugated (Amersham Pharmacia, Piscataway, NJ, USA) and immunoreactive proteins were visualized with chemiluminescence using SuperSignal WestPico Chemiluminescent Substrate (Pierce, Rockford, IL, USA).

Immunocomplexes were quantified by a densitometric semiquantitative analysis using NIH ImageJ software (NIH, Rockville Pike Bethesda, MA, USA). The densitometric quantification of the targeted proteins were normalized by the densitometric quantification of the housekeeping protein observed in the same membrane.

### HO-1 protein level

HO-1 was detected in cell lysates by Immunometric Enzyme-Linked Immunosorbent Assay (ELISA) kit (HO-1 human, ELISA development set, Enzo Life Sciences, Farmingdale, NY, USA) according to the indications of the producer. Briefly, samples were plated and incubated with HO-1 capture antibody followed by washing steps and the addition of detection antibody for HO-1. After the second washing steps the plate was incubated with streptavidin conjugated to horseradish peroxidase and washed once again. The plate was thereby incubated with TMB substrate and finally blocked with HCl 1N. After blanking the plate reader against the substrate, optical density was read at 450nm. Values of HO-1 concentration were draw from the standard tuition and expressed in ng/ml.

### Lipid peroxidation

Lipid peroxidation was evaluated in plasma samples in terms of malondialdehyde production malondialdehyde production using the commercially available Lipid Peroxidation (MDA) Assay Kit nr. ab118970 (Abcam, Cambridge, UK). The colorimetric reaction was measured with the Labsystem Multiskan MS plate reader (LabX Midland, ON, CA) at an optical density of 540nm and malondialdehyde production was expressed as nmol/mL.

### cAMP measurement

cAMP levels were evaluated in plasma samples using the ELISA kit nr. KA3388 (Abnova, Neihu District. Taipei City, TW). To perform the analysis on plasma samples we used the acetylation protocol according to manufacturer instructions. Once obtained the colored reaction, the plate was read in a microplate reader at 450nm wavelength. After making the correction for the dilution of the sample, the concentration of cAMP was calculated using a built-in 4PLC software (Multiskan FC microplate photometer, Thermofisher, Scientific Waltham, Massachusetts, USA).

### Echocardiography

LV mass was assessed by echocardiography after enrollment in the study and interpreted off-line by an experienced operator blinded to patient identity/status. LV mass was measured at M-mode echocradiography normalizing for body surface area and normal values were defined according to guidelines: <116 g/m^2^ for males and <96 g/m^2^ for females [17].

### Statistical analysis

Data were evaluated on a Mac mini (Apple Computer, Cupertino, CA, USA) using a GraphPad Prism 5.0 software (LA Jolla, CA, USA). Data are expressed as mean ± SEM. The normal distribution of the variables was formally verified beforehand by Kolmogorov-Smirnov test and the variables that did not meet this criterion (Mypt-1 and phosphorylated-Mypt-1) underwent logarithmic transformation and retested for Gaussian distribution, therefore were appropriately analyzed by means of t-test. To determine statistical correlation among different factors we used the rho Spearman’s correlation coefficient. Values at 5% level or less (p<0.05) were considered significant.

## Results

The results of the patients under antihypertensive therapy (N = 3) were compared with those of the other patients of the cohort (N = 7) finding no difference between them, therefore all subjects were considered in the same analysis. No statistical significance was observed for the comparison of the variables of Table 2 between the patients with Fabry disease and healthy subjects.

### LV mass

Cardiac LV mass in male Fabry disease patients was higher (123.72 g/m^2^ ± 2.03) compared to normal values defined according to guidelines (<116 g/m^2^) [17]. In Fabry disease female patients, cardiac LV mass was also higher, (132.09 g/m^2^ ± 6.72) compared to the normal values (<96 g/m^2^) [17].

### p22^phox^ protein expression

**Fig 1:**
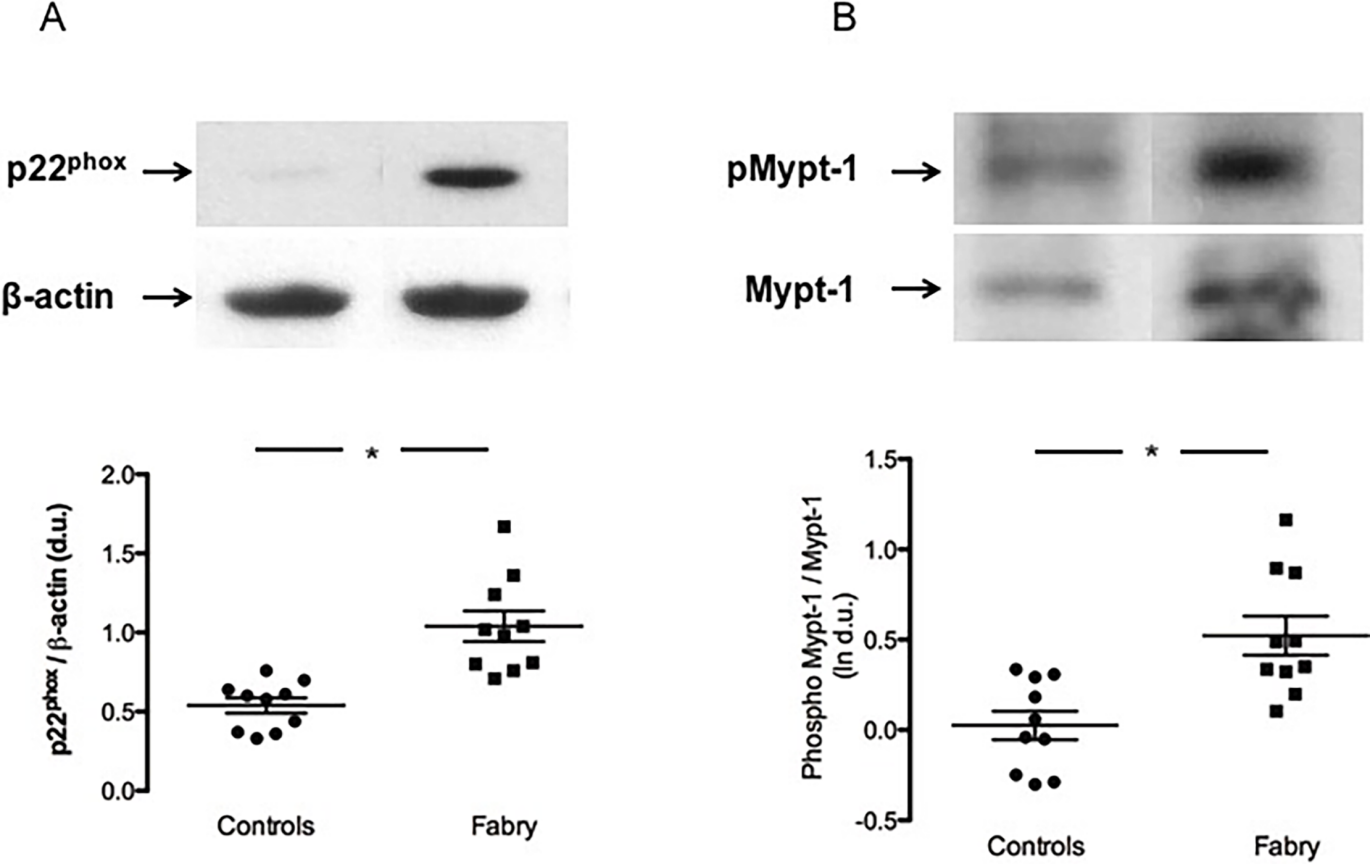
panel A: p22^phox^ protein expression. Representative Western blot of protein lysates harvested from healthy subjects (Controls) and patients (Fabry), (Top of the figure). Densitometric analysis of Western blot products showing the higher expression of p22^phox^ in Fabry patients, compared to healthy subjects. Data are shown ±SEM. *: *p*<0.01. panel B: MYPT-1 phosphorylation state. Representative Western blot of phosphorylated MYPT-1 and MYPT-1 (Top of the figure). The densitometric analysis, expressed as ln of d.u., shows higher phosphorylation of MYPT-1 in Fabry patients compared to healthy subjects. Data are shown ±SEM. *: *p*<0.01.

As shown in Fig 1, panel A, the evaluation of p22^phox^ protein expression exhibited a significant difference in Fabry patients compared to healthy subjects. p22^phox^ was, in fact, highly expressed in Fabry patients compared to healthy subject: 1.04±0.09 d.u. vs 0.54±0.05 d.u. p<0.01.

### MYPT-1 phosphorylation state

The assessment of Rho kinase activation in terms of MYPT-1 phosphorylation is shown in Fig 1, panel B. MYPT-1 phosphorylation state (ln) was significantly higher in Fabry patients compared to healthy subjects: 0.52±0.11 d.u. vs 0.03±0.08 d.u., p<0.01.

### ERK 1/2 phosphorylation state

**Fig 2:**
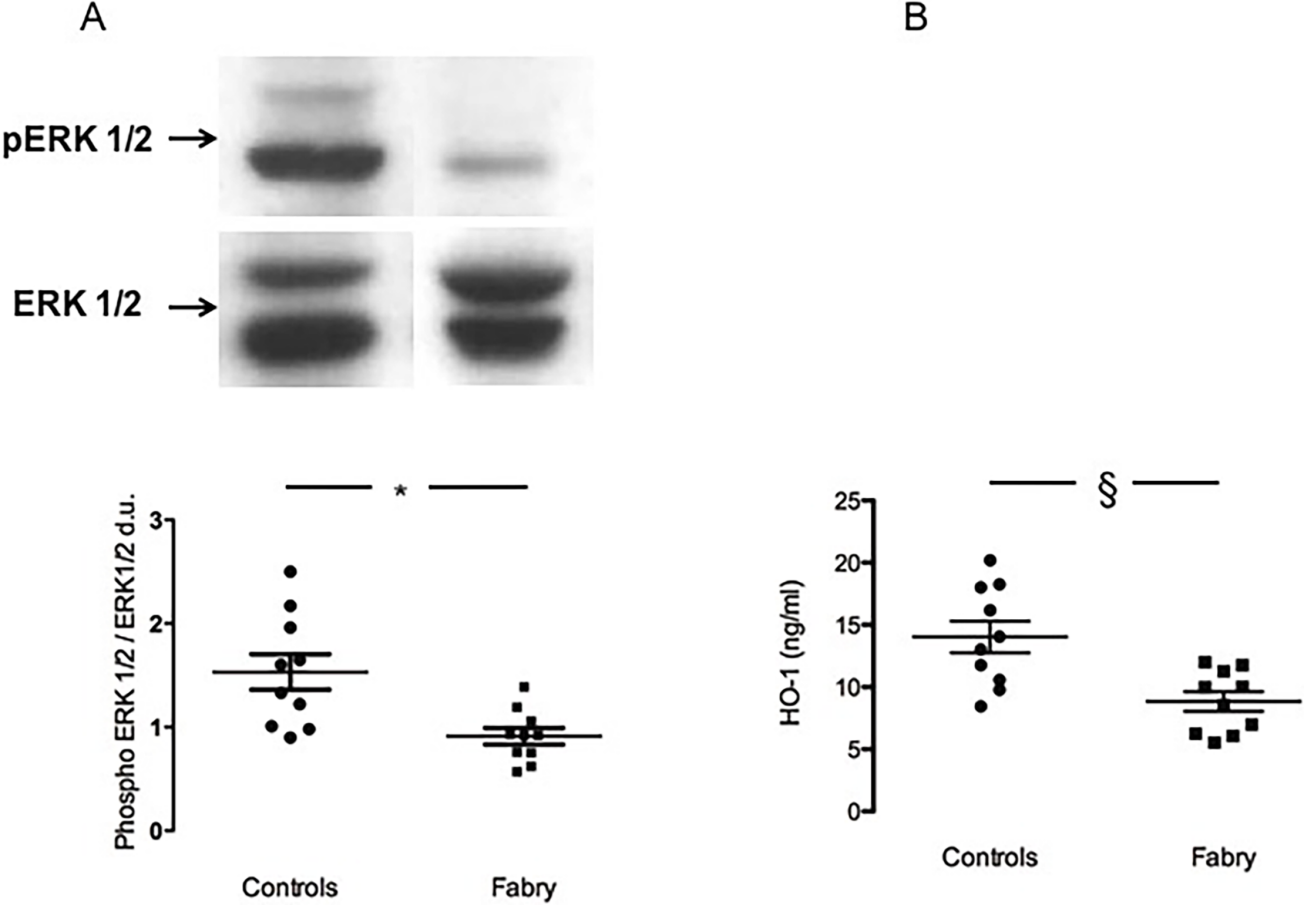
panel A: ERK 1/2 phosphorylation state. The top of the figure shows representative ERK 1/2 phosphorylation status in Fabry patients (Fabry) and in healthy subjects (Controls). Data are shown ±SEM. *: *p* = 0.004. panel B: HO-1 protein expression. HO-1 protein concentration (ng/mL). Data are shown ±SEM. §: *p*<0.02.

Contrary to our expectation, the phosphorylation state of ERK 1/2, was significantly decreased in Fabry patients compared to healthy controls: 0.91±0.08 d.u. vs 1.53±0.17 d.u., p = 0.004 (Fig 2, panel A).

### HO-1 protein level

HO-1 protein concentration was measured as antioxidant power in Fabry disease and resulted to be significantly less induced in Fabry patients compared to healthy controls: 8.84±0.79 vs 14.03±1.23 ng/mL, p<0.02 (Fig 2, panel B)

Looking at the presence of a relationship between LVH and MYPT-1 phosphorylation and p22^phox^ protein expression, no correlation was found: for LVH and MYPT-1 phosphorylation r = 0.1571, p = 0.50, n.s. and for LVH and p22^phox^ protein expression r = 0.05476, p = 0.65, n.s.

### Lipid peroxidation

**Fig 3:**
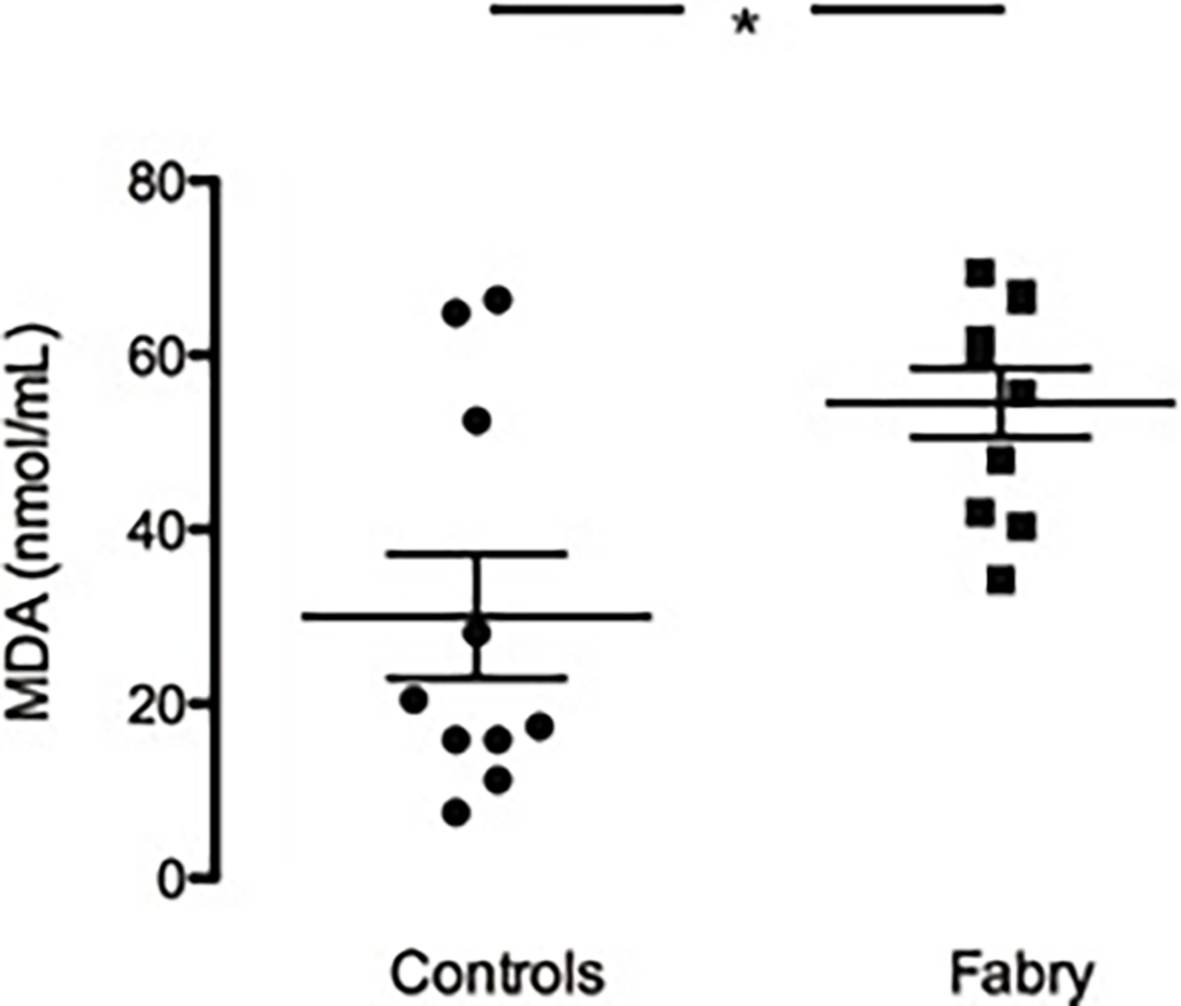
Lipid peroxidation state. Colorimetric analysis of MDA concentration (nmol/mL) in Fabry subjects (Fabry) compared to healthy subjects (Controls). Data are shown ±SEM. *: *p* = 0.01.

As shown in Fig 3, MDA concentration was significantly higher in plasma of Fabry patients compared to healthy controls (54.51±3.97 vs 30.05±7.11 nmol/mL p = 0.01).

No correlation was found between the MDA production and the markers of OxSt and proteins related to OxSt considered in the present study: MDA and p22^phox^ (r = 0.036, p = 0.96); MDA and HO-1 (r = 0.133,p = 0.73); MDA and phosphoMYPT-1 (r = 0.22; p = 0.60). In addition, no correlation was found between MDA production and left ventricular mass in Fabry patients (r = 0.55, p = 0.33).

### cAMP levels

**Fig 4:**
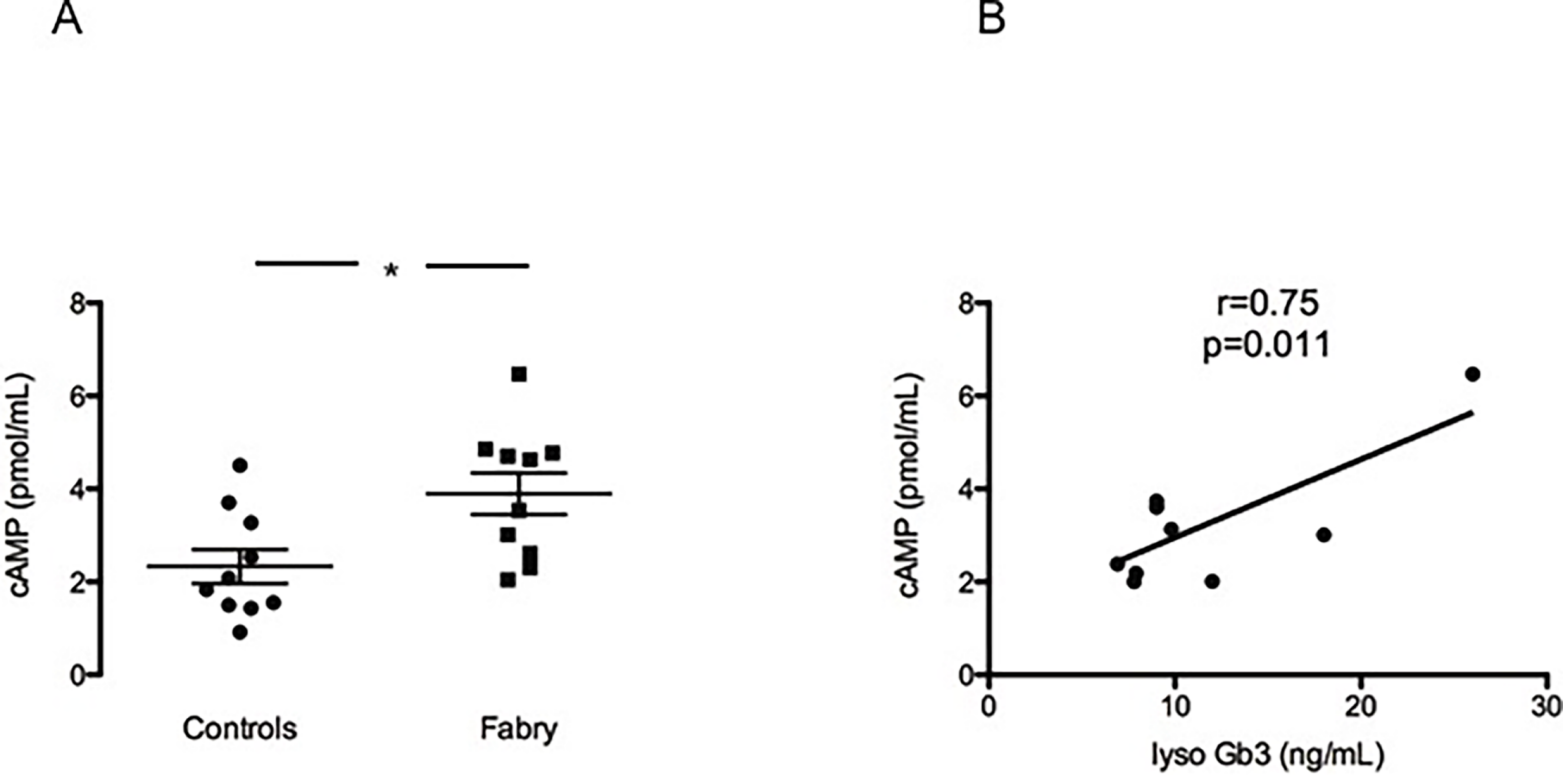
cAMP levels and correlation with LysoGb3. Panel A shows cAMP levels (pmol/mL) in Fabry subjects (Fabry) compared to controls (Controls) Data are shown ±SEM. *: *p* = 0.01. In panel B there is a positive correlation between cAMP levels in Fabry patients and their LysoGb3 levels. r = 0.75, p = 0.011.

The cAMP levels in plasma samples from Fabry patients were significantly higher compared to the levels found in healthy controls (3.89±0.44 vs 2.33±0.36 pmol/mL p = 0.01) Fig 4, panel A. Moreover, the relationship between cAMP levels and LysoGb3 levels has been evaluated by performing a linear regression analysis. The higher cAMP levels were significantly correlated to the higher LysoGb3 levels in Fabry patients (r = 0.75, p = 0.01) as it is shown in Fig 4, panel B.

## Discussion

Given the close relationship between OxSt and cardiovascular-renal remodeling, that studies from our laboratory have demonstrated using markers of OxSt and proteins related to OxSt such as p22^phox^, lipoperoxides and RhoA/Rho kinase pathway [14,18], we thought it would be of interest to examine the status of OxSt in patients with Fabry disease and its relationship to proliferative/remodeling processes at cardiovascular level.

The results of this study show that OxSt is clearly present and active in Fabry patients and that the clinical linkage between the markers OxSt and cardiac remodeling in the patients considered in this study has been documented at the level of association. No correlation, in fact, was found in Fabry patients between LV mass and any marker of OxSt used. This failure to show a correlation could perhaps be attributed to the limited number of subjects we have enrolled due to the rare nature of the disease. However, cardiac remodeling in terms of LV mass is clearly evidenced in our Fabry patients.

Gb3 accumulation, a hallmark of Fabry disease, is not limited to the lysosomes and is, in fact, also increased in the plasma membranes and caveolae of endothelial cells. It has recently been suggested that Gb3 accumulation in the endothelium is able to dysregulate the activity of endothelial NO synthase (eNOS) and may compromise caveolar stability and the downstream signal transduction of caveolar proteins such as eNOS [19]. eNOS dysregulation, which specifically reduces NO production, would favor increased OxSt with the increased levels of reactive oxygen species being central to the mechanism of Fabry disease cardiovascular disease. In fact, α-GalA knockdown human endothelial cell line by RNA interference showed a dramatically enhanced 3-nitrotyrosine (3NT) production in addition to Gb3 accumulation and reduced eNOS activity. The 3NT elevation is a result of a post-translational modification of proteins that occurs in situations of increased OxSt caused by reduced NO bioavailability [19]. Moreover, the excessive production of superoxide and other ROS reduces NO bioavailability via their chemical reaction with NO to produce peroxynitrites. This induces OxSt, cell damage via lipid peroxidation, inactivates enzymes and other proteins by oxidation and nitration.

NADPH oxidases (NOXs) are a group of membrane-associated enzymes that play a major role in the fine tune of redox state in the cells. These enzymes catalyze the production of ROS by the association of different membrane and cytosolic subunits. Among NOX family, the NOX2 isoform requires the association of p22^phox^ and other three cytosolic subunits to be activated: once these subunits translocate to the membrane via a specific regulated process, a conformational change of the complex promote the electron transfer from NADPH to molecular oxygen (O_2_) to form superoxide (O_2_^−^) which triggers the induction of free radicals, peroxinitrites and other ROS [20]. Our data show that in Fabry patients p22^phox^ is overexpressed compared to healthy subjects, suggestive of OxSt which likely elevates ROS and thereby OxSt related signaling. The increased production of oxidants may impact on the lipidic components of cellular membranes and affects the lipid signaling mediators. During the free radicals induced lipid peroxidation, in fact, the balance between lipids disruption and repair capacity is altered thus inducing inflammation, apoptosis and atherogenesis. Alongside the lipid hydroperoxides (LOOH), primary products of peroxidation, different aldehydes can be formed as secondary products such as malondialdehyde (MDA), propanal, hexanal, and 4-hydroxynonenal (4-HNE) [21]. Recently, it has been shown that Fabry patients before ERT had higher lipid peroxidation levels, which could not be reversed by the treatment [22]. In our study the increased MDA concentration in Fabry disease is confirmed, suggesting that in Fabry disease oxidative damage impacts on lipid structures contributing in the long term to the onset of pathological processes leading to cardiovascular-renal and cerebrovascular remodeling.

The activation of RhoA/Rho kinase pathway is recognized to be related with OxSt and involved in atherosclerosis and cardiovascular-renal remodeling [8,14]. Rho kinase pathway promotes Ca^2+^ sensitization of smooth muscle contraction and the modulation of myosin light chain phosphorylation through the inhibition, via an inhibiting phosphorylation, of MYPT-1 phosphatase activity. In the present study, we demonstrate that the Rho kinase pathway is activated in Fabry patients via the significantly higher phosphorylation status of MYPT-1 in these patients compared to healthy subjects. The haemodynamic and structural changes induced by Rho kinase activation has been documented in CKD and dialysis patients [23] and its activation also in Fabry patients suggests that Rho kinase pathway activation may play a major role in the status of the cardiovascular and renal remodeling of these patients.

The intracellular signaling mediated by ERK1/2 is likewise closely linked to OxSt. It represents an important effector for the hypertrophic response via phosphorylation of nuclear targets (e.g., the c-myc, the anti-apoptotic c-jun and ATF2). The activated transcription factors lead to transcriptional reprogramming and altered gene expression associated with hypertrophy [24]. Indeed, ERK1/2 signaling can mediate both differentiation and proliferation. While an increase in ERK1/2 phosphorylation was expected in a population with high risk for cardiovascular-renal remodeling such as Fabry patients, surprisingly, we have found that the phosphorylation state of ERK1/2 was reduced in our Fabry patients compared to healthy subjects. However, high LysoGb3 has been recently shown in an in vitro model to be associated with increased intracellular cAMP. This is of importance as in cardiac myocytes, the activation of ERK1/2 is under cAMP control and elevated cAMP may induce a reduction in the phosphorylation of ERK1/2 via PKA activity [17,18]. The upstream inducers of MAPKs family are extracellular stimuli converging on GTPase proteins as Ras and Rap1, which, once activated, trigger a phosphorylation cascade to switch on ERK1/2. PKA via stimulation of Rap1 antagonizes Ras by capturing Raf1 from the complex, reducing therefore ERK1/2 and its phosphorylation [15]. The increased cAMP plasma level of our Fabry patients and its positive correlation with Fabry LysoGb3 plasma levels gives the rationale to the reduced ERK1/2 phosphorylation in addition to confirm in humans the relationship between LysoGb3 and cAMP pathway previously demonstrated in an “in vitro” model.

HO-1, an antioxidant defense enzyme induced by OxSt was significantly reduced compared controls. HO-1 acts on heme producing CO and biliverdin which is further metabolized to bilirubin, a potent antioxidant itself thus contributing to regulation of vascular tone and to endothelial function; in the long term, HO-1 has also been shown to have antiinflammatory and antiproliferative effects [12,25]. There are three different isoforms of HO: HO-1, HO-2 and HO-3. HO-1 has a very low basal expression, but it increases rapidly upon OxSt and NO stimulation, while HO-2 and HO-3 are constitutively expressed [12]. The HO-1 fine tuning depends on both NO and ERK 1/2 and this fact could provide a mechanistic rationale for our findings. Indeed, the accumulation of Gb3 strongly influences the NO production via dysregulation of eNOS activity. Thus the reduced circulating levels of NO might act in such a manner to reduce the signal for HO-1 expression. Moreover, it is reported that HO-1 activity serves to counterbalance overproduction of superoxide via ERK1/2 activated NADPH oxidases [12]. Thus, in addition to the reduced levels of NO, the reduced ERK1/2 activation found in Fabry disease would also likely contribute to reduced HO-1 induction. This suggests that Fabry patients do not react normally to OxSt and this might provide an explanation as to why ERT does not completely halt or reverse by itself the progression of Fabry disease.

## Conclusion

In conclusion, this study demonstrates that OxSt occurs in Fabry disease and the reaction to OxSt in terms of antioxidant defenses such as activation of HO-1, is altered in Fabry patients. This dysregulation may play an important role through ROCK signaling induced by OxSt in the cardiovascular-renal remodeling.

The extrapolation of the data obtained in circulating mononuclear cells to mechanisms that should take place in myocardial cells could be a limitation of this study. However, it should be noted that circulating blood cells are a valid useful tool in vascular biology to study ‘ex-vivo’ pathophysiological mechanisms of hypertension and remodeling [26]. In addition, it has been increasingly recognized the role of various inflammatory mechanisms such as mononuclear leukocyte infiltration (polymorphonuclear and mononuclear cells) and intracellular OxSt for the development of hypertensive target organ damage [26,27]. Furthermore, leukocyte ROCK has been shown to increase the leukocyte infiltration into the vascular wall resulting in the proinflammatory cytokines [28] and an increase in ROCK activity in peripheral leukocytes has been shown in patients with hypertension [29].

The findings provided by this study add further information in the pathophysiology of Fabry disease, although, given the small size of patients studied, an unavoidable consequence of the rare nature of Fabry disease, our conclusions should be considered preliminary. In fact, if on one hand this study being, to our knowledge, the first which uses a molecular biology approach to investigate the presence of OxSt in Fabry disease, might justify the limited number of the patients enrolled, on the other hand the results of this study could serve as a useful working hypothesis for further studies with a larger number of patients enrolled as well as to compare Fabry patients treated and not treated with ERT in order to evaluate and more conclusively demonstrate the role of OxSt and OxSt-induced inflammation in the cardiovascular-renal remodeling of Fabry disease. Moreover, a better understanding of the specific molecular signaling response to OxSt in Fabry disease could suggest additional interventions by either pharmacological or nutritional measures aimed at halting the progress of cardiac and cerebrovascular disease, and nephropathy that occur in Fabry patients via the reduction of oxidative stress.

## Supporting information

S1 File Data of every single patient for each parameter considered in the study. (XLSX)
